# The Influence of Zearalenone on Selected Hemostatic Parameters in Sexually Immature Gilts

**DOI:** 10.3390/toxins13090625

**Published:** 2021-09-06

**Authors:** Ewa Jakimiuk, Justyna Radwińska, Maciej Woźny, Andrzej Pomianowski, Paweł Brzuzan, Paweł Wojtacha, Kazimierz Obremski, Łukasz Zielonka

**Affiliations:** 1 Department of Veterinary Prevention and Feed Hygiene, Faculty of Veterinary Medicine, University of Warmia and Mazury in Olsztyn, Oczapowskiego 13/29, 10-718 Olsztyn, Poland; kazimierz.obremski@uwm.edu.pl (K.O.); lukasz.zielonka@uwm.edu.pl (Ł.Z.); 2 Department of Internal Diseases with Clinic, Faculty of Veterinary Medicine, University of Warmia and Mazury in Olsztyn, Oczapowskiego 14, 10-718 Olsztyn, Poland; justyna.radwinska@uwm.edu.pl (J.R.); andrzej.pomianowski@uwm.edu.pl (A.P.); 3 Department of Environmental Biotechnology, Faculty of Geoengineering, University of Warmia and Mazury in Olsztyn, Słoneczna 45G, 10-709 Olsztyn, Poland; brzuzan@uwm.edu.pl; 4 Department of Industrial and Food Microbiology, Faculty of Food Science, University of Warmia and Mazury in Olsztyn, Plac Cieszynski 1, 10-726 Olsztyn, Poland; pawel.wojtacha@uwm.edu.pl

**Keywords:** blood coagulation, catalase, endothelial cells, nitric oxide, vascular toxicity, von Willebrand factor, zearalenone

## Abstract

Vascular toxicity induced by xenobiotics is associated with dysfunctions or damage to endothelial cells, changes in vascular permeability or dysregulation of the vascular redox state. The aim of this study was to determine whether per os administration of zearalenone (ZEN) influences selected hemostatic parameters in prepubertal gilts. This study was performed on female gilts divided into a control group which received placebo and an experimental group which received ZEN at a dose of 5.0 µg·kg^−1^ b.w. × day^−1^. On days 14, 28 and 42, blood samples were collected from the animals for analyses of hematological, coagulation and fibrinolysis parameters, nitric oxide, von Willebrand factor antigen content and catalase activity. The results demonstrated that the treatment of gilts with ZEN at a dose below no observable adverse effect level did not affect the primary hemostasis and the blood coagulation cascade. However, ZEN could have temporarily affected the selected indicators of endothelial cell function (increase of von Willebrand factor, decrease of nitric oxide levels) and the oxidative status plasma (decrease of catalase activity) of the exposed gilts. In summary, these results suggest that the adaptive response to ZEN-exposure can induce a transient imbalance in the vascular system by acting on vascular endothelial cells.

## 1. Introduction

The components of circulatory and hemostatic systems are particularly vulnerable to environmental substances (xenobiotics) [1,2,3,4]. Vascular toxicity induced by chemical compounds is associated mainly with dysfunctions or damage to endothelial cells, changes in vascular permeability or dysregulation of the vascular redox state [5]. The presence of estrogen receptors (ERs) and other nuclear receptors sensitive to steroids and xenobiotics in the hemostatic system components indicates that both endogenous estrogens and endocrine-disrupting chemicals can modulate hemostatic functions [3,4,6,7,8]. In females, endogenous estrogens exert protective effects on vascular wall components and modulate the inflammatory response and the function of immune cells [9,10]. The interactions between hemostatic and immune system components and factors capable of disrupting these parameters play a particularly important role in the hemostatic system [11]. The hormonal status of females is modified not only during the reproductive cycle or estrus, but also by exposure to environmental compounds with estrogenic properties [12].

Zearalenone (ZEN) is a secondary metabolite of mold fungi of the genus *Fusarium*. Consumption of contaminated grains and cereal products is the main source of exposure to this mycotoxin [13]. ZEN disrupts endocrine functions in the body by interacting with steroid receptors and influencing steroidogenic enzymes [14]. This metabolite also interacts with transcription factors other than ERs and modulates the expression of numerous genes [15,16]. The duration and intensity of ZEN’s impact on the body varies subject to the period of exposure, the ingested dose and the species-specific biotransformation pathways [17,18]. Research on ZEN’s toxicity indicates that among livestock species, pigs (in particular prepubertal female gilts) are most sensitive to the estrogenic effects of ZEN [13,17,19]. The mechanism of ZEN activity at the cellular and tissue level, in particular during exposure to “non-estrogenic” doses of the mycotoxin below no observable adverse effect level (NOAEL), can compromise animal performance and/or health by modifying the redox status and disrupting immune function. Intoxication with ZEN leads to the generation of reactive oxygen species (ROS) and/or inhibits the expression of antioxidant factors [20,21]. Oxidative stress and the accompanying increase in ROS levels can modulate the activity of estrogens and estrogen-like compounds by influencing ER expression, other transcription factors and, consequently, their biological activity [22]. ZEN exerts immunotoxic effects at high doses as well as immunomodulatory effects, and its proinflammatory or anti-inflammatory properties may be manifested depending on the targeted tissues [23,24,25,26].

Selected ZEN metabolites have been most extensively researched when investigating the impact of mycoestrogens on the cardiovascular system. The protective effect of α-zearalanol on endothelial cells has been evaluated both in vivo and in vitro [27,28,29,30,31]. Figtree et al. [32] reported that another ZEN metabolite, zearalanone, induced changes in arterial vascular tone (relaxation) in rabbits independently of endothelial cells and estrogen receptors. An in vivo study by Altavilla et al. [33] demonstrated that α-zearalenol exerts protective effects on the circulatory system via ERs, by stimulating the activity of endothelial nitric oxide synthase (eNOS) and nitric oxide (NO) secretion.

ZEN’s influence on the vascular and/or hemostatic system has been rarely studied. Studies on the influence of ZEN-induced mycotoxicosis on animal health and productivity have demonstrated that ZEN can modulate selected components of hemostasis. The conclusions regarding ZEN’s adverse effects on the coagulation system have been derived largely by extrapolating the results of in vivo and in vitro studies investigating the hepatotoxicity and/or hematotoxicity of high ZEN doses [34,35]. ZEN also exerted hematotoxic effects on the parameters of red and white blood cells and blood platelets [36,37]. Woźny et al. [38] observed changes in coagulation parameters (bleeding) in trout exposed to ZEN. In rats, ZEN exposure disrupted retinal microcirculation and increased the risk of retinopathy [13]. Kitambi et al. [39] demonstrated that ZEN induced selective changes only in the intraocular vasculature of zebrafish. In a study by Lee et al. [40], ZEN (30 µM) stimulated apoptosis in bovine aortic endothelial cells independently of ERs or ROS production. According to a recent study by Güner [5], ZEN causes vascular irritation in a dose-dependent manner.

Vascular integrity, the number and function of blood platelets and the plasma coagulation system are responsible for the proper function of the hemostatic system. Screening and/or specialist tests are performed to evaluate the hemostatic system based on the observed symptoms. Blood platelets and coagulation parameters are indicators of primary and secondary hemostasis, respectively [41]. Under physiological conditions, blood platelets are maintained in an inactive state by mediators (such as NO), but proinflammatory cytokines and endothelial cell dysfunctions can activate blood platelets and disrupt the balance between hemostatic and immune systems [11]. Vascular reactivity is largely determined by the secretory activity of the endothelium. The vascular bed is lined with endothelial cells which are the first cells to come into contact with xenobiotics entering the blood stream. In addition to regulating vascular tone, vascular permeability and clotting, endothelial cells also play an important role in adaptive and innate immune responses [42]. In blood vessels, endothelial activity is mediated mainly by NO, which dilates vessels, prevents blood cells from aggregating and adhering to the surface of endothelial cells and prevents leukocyte adhesion and penetration [43]. The von Willebrand factor (vWf) plays an important role in angiogenesis, cell proliferation and apoptosis, inflammation and cancer. This plasma protein participates in coagulation, and changes in its concentration or quality are manifested by extravasations or bleeding on the surface of skin and mucous membranes [44]. As the secondary antioxidant enzyme in the peroxidative defense system, catalase is most active in erythrocytes and hepatocytes, but its activity is also modified in the blood serum [45,46,47]. Serum catalase protects vascular endothelial cells against damage induced by H_2_O_2_ [48]. A gradual decrease in serum catalase activity can be indicative of vascular disease [49,50].

The effects of ZEN intoxication on hemostasis have not been directly evaluated to date. The maximum ZEN limits for piglet feeds (0.1 mg·kg·feed^−1^) and NOAEL values for piglets (10.4 µg·ZEN·kg^−1^ b.w. × day^−1^) [13,16,51] have been adopted by the European Union to minimize the risk of hyperestrogenism in the reproductive system. However, these limits do not eliminate ZEN’s adverse effects on other tissues and organs [52]. The vascular system is highly susceptible to ZEN because it contains ERs as well as other receptors and enzymes that regulate the biotransformation of xenobiotics. Due to physiological interactions between hemostatic and immune system components, the vascular system is also sensitive to compounds with immunomodulatory and pro-oxidative properties. In view of the properties of ZEN and its mode of action, the aim of this study was to determine whether exposure to a ZEN dose of 5.0 µg·kg^−1^ b.w. × day^−1^ for 42 days influences selected hemostatic parameters in gilts.

## 2. Results

The results of the hematological analysis of the prepubertal gilts from the control group (placebo) and the group exposed to ZEN (at 5.0 µg·kg b.w.^−1^ × day^−1^) on days 14, 28 and 42 of the experiment are presented in Table 1. The platelet count and the mean platelet volume did not differ significantly between any of the experimental groups (*p* > 0.05, Kruskal‒Wallis test).

On days 14, 28 and 42 of the experiment, most measurements of blood coagulation were fairly similar in the control and ZEN-treated groups (Table 2). However, differences in thrombin time values between the experimental groups were suggestive. Note that, even though the differences were not statistically significant (*p* = 0.072, Kruskal‒Wallis), all the thrombin time values in the ZEN-treated group were lower than those in the control group on days 14 and 28.

In the blood plasma of the pigs, the concentrations of von Willebrand factor and NO, as well as the catalase activity, differed significantly between groups, measurement times or both (*p* < 0.05, Kruskal‒Wallis; Table 3). In the ZEN-treated group, the von Willebrand factor concentration decreased significantly from day 14 to day 42 of the experiment (*p* = 0.033, Dunn’s post hoc test), whereas in the control group, the concentrations were similar throughout the experiment. Although the difference between the ZEN-treated group and the control group on day 42 was not statistically significant (*p* > 0.05; Dunn’s), all the values in the ZEN-treated group were lower than those in the control group. NO concentrations did not differ in a statistically significant manner between particular experimental groups (*p* > 0.05; Dunn’s). However, it is interesting to note that there appears to be a downward trend with time in both groups, with concentrations starting around 190 µmol∙L^−1^ and ending around 145 µmol∙L^−1^. Moreover, the values in the ZEN-treated group appeared to drop faster than those in the control group, reaching 130.2 µmol∙L^−1^ on day 28 when those in the control group were 172.2 µmol∙L^−1^ (Table 3). Finally, plasma catalase activity in the control gilts was significantly higher on days 28 and 42 than on day 14 (*p* < 0.05; Dunn’s), whereas these values remained about the same in the ZEN-treated gilts. Note that, although the differences were not statistically significant, all the values in the ZEN-treated group were higher than those in the control group on day 14, but the opposite was true on days 28 and 42.

## 3. Discussion

This is the first in vivo study to directly evaluate the influence of ZEN on blood clotting and the vascular endothelium. The main limitations of this study were its small sample size and the fact that only selected hemostatic parameters were analyzed. Although these limitations can reduce statistical power to detect differences between the experimental groups, we believe that this study provides new information, making it a valuable contribution to the latest in vitro research into ZEN’s effect on endothelial cells and the vascular system [5,40,53,54]. The present findings suggest that prolonged exposure to ZEN, even at doses below NOAEL values, can affect the porcine vascular system.

According to research, ZEN’s hematotoxicity affects blood clotting [55]. Previous studies have demonstrated that ZEN exerts hematotoxic effects in a dose-dependent manner. In studies of rats [36], mice [56] and pigs [57], platelet counts decreased with an increase in ZEN dose. Platelet counts and other blood cell components also decreased in pigs exposed to ZEN in vitro [58]. In the current study, not only did platelet counts not decrease in the experimental gilts exposed to ZEN doses below NOAEL values but were even higher (days 14 and 28; Table 1), although they did not exceed the upper reference limit for pigs. However, previous research indicates that components of the hemostatic system participate in the inflammatory response [11,44,59]; thus, it is worthwhile to further investigate the possible immunomodulatory effects of ZEN. Platelets link coagulation processes with inflammatory responses [60]. Platelet counts increase during secondary/reactive thrombocytosis, which is a normal response to inflammation. Cytokines, including IL-1, IL-3 and IL-6, play pivotal roles in crosstalk between inflammation and coagulation [61,62,63]. Previous research indicates that ZEN can affect the secretion of the above factors [23,64,65,66]. However, so far, the effect of cytokines on the vascular system has not been linked to ZEN exposure.

With most of the blood coagulation indicators examined in our study, there was little difference between groups or over time. However, the exception was thrombin time; although the differences were not statistically significant, the results suggested that these values may be lower in ZEN-treated gilts than in control gilts after 14 and 28 days of ZEN exposure (Table 2). Thrombin time measures the time it takes for soluble fibrinogen to be converted into insoluble fibrin, which forms a hemostatic clot. Thrombin time is dependent on fibrinogen concentration, but it is not affected by activation of the external and internal coagulation pathways. Thus, the drop in thrombin time that was observed does not indicate coagulation disorders; instead, it suggests an increase in fibrinogen concentrations, e.g., during pregnancy or compensatory synthesis of proteins in the liver. Therefore, it can be generally concluded that a comparison of the coagulograms of the control and treated pigs suggests that ZEN (administered at a dose below NOAEL values) did not disrupt the synthesis of coagulation factors in the liver or the coagulation cascade.

Until recently, changes in von Willebrand factor content have been linked with hemorrhagic diathesis, but changes in its content are increasingly regarded as an indicator of inflammation and dysfunction of the vascular endothelium [44,67]. In our study, the plasma concentration of vWf in ZEN-treated gilts was significantly lower after 42 days of the experiment than after 14 days (Table 3). This decrease suggests that ZEN exposure could inhibit the release of this factor from endothelial cells. The secretion of vWf is regulated by estrogens, inflammatory mediators and ROS [68]. Estrogens stimulate the proliferation of endothelial cells and the production and release of von Willebrand factor via ERs in a dose-dependent manner [69]. The influence of oxidative stress on von Willebrand factor release seems to be determined by the type of ROS: the superoxide anion stimulates the release of vWf from the endothelium, whereas H_2_O_2_ inhibits vWf secretion in a dose-dependent manner [68]. To the best of our knowledge, this is the first study to analyze ZEN’s influence on von Willebrand factor. The observed decrease in the factor secretion could be linked to changes in catalase activity (Table 3), which possibly affected the H_2_O_2_ concentration. Our observations are in agreement with previous research in which ZEN exposure increased leukocyte counts (including neutrophils), which could have also increased the H_2_O_2_ concentration [56,70].

Endothelial cell dysfunction is associated with decreased synthesis or greater inactivation of NO. Estrogens stimulate the release of NO from endothelial cells, but their influence on other nitric oxide synthase (NOS) isoforms can be tissue-specific [71,72,73]. In this study, an interesting downward trend with time in NO concentration was observed in both groups, and the values in the ZEN-treated group appeared to drop faster than those in the control group (Table 3). Lee et al. [53] found that ZEN decreased the expression of endothelial nitric oxide synthase (eNOS) protein and suppressed NO production in bovine aortic endothelial cells independently of ERs. The expression of eNOS mRNA and/or protein was not examined in this study; therefore, ZEN’s effect on porcine NO synthesis cannot be evaluated. Oxidative stress also affects eNOS activity and the bioavailability of NO to the endothelium [73,74]. The activation of proinflammatory transcription factors changes the profile of ROS (↑superoxide radical/↓H_2_O_2_) produced by NADPH oxidases, which can decrease the availability of NO to endothelial cells [75,76,77]. In view of the stimulatory influence of estrogens and the inhibitory effect of oxidative stress on NO release [43], it appears that ZEN’s oxidative potential may outweigh its estrogenic properties. It is possible that ZEN-induced oxidative stress and/or increased ROS production could temporarily inactivate NO and compromise the vascular relaxation response [53,78]. This suggests that such changes in NO content or bioavailability in blood vessels could cause intensified bleeding in vivo (blood sampling, slaughter), particularly during the first 2–3 weeks of ZEN exposure.

In our study, plasma catalase activity in the control group increased over time, whereas its activity remained relatively stable in the ZEN-treated group (Table 3). The increase in the control animals could indicate age-related changes in the concentration of H_2_O_2_, which modulated the expression of catalase mRNA via a feedback mechanism [45,79,80,81]. Catalase activity was the only marker of oxidative stress that was analyzed in the present study, but in vivo studies conducted by Marin et al. [23], Shi et al. [82] and Cheng et al. [83] demonstrated that oxidative stress can be induced at a wide range of ZEN concentrations. Catalase plays an important role in erythrocyte metabolism, and its levels usually increase in the plasma due to leakage from damaged red blood cells. However, the hematological indicators in the ZEN-exposed gilts in the present study did not differ significantly between any of the experimental groups (Table 1). Previous studies have demonstrated that ZEN exposure increases granulocyte counts, particularly those of neutrophils, which can increase H_2_O_2_ levels [84,85]. In the present study, catalase activity decreased on day 28 in the ZEN-exposed group. Similarly, Liu et al. [86] also observed that exposure to ZEN at a dose of 1.04 mg·kg·feed^−1^ for 35 days decreased catalase activity and increased ROS production in the intestinal wall of gilts. Bellanti et al. [87], Michałek et al. [45] and Salah-Abbès et al. [88] linked a decrease in catalase activity with oxidative stress and the depletion of defense mechanisms against ROS. ZEN can induce oxidative stress via various mechanisms in a manner that is dependent on the dose, the duration of exposure and the targeted tissues [47,89,90,91]. According to Grzybowski [92], a drop in catalase activity is also influenced by interleukin 1β. Marin et al. [23] and Jia et al. [20] reported that ZEN stimulated the secretion of proinflammatory cytokines, including interleukin 1β. In summary, these results suggest that the adaptive response to ZEN exposure can influence the natural increase over time in plasma catalase activity that was observed in control gilts.

## 4. Conclusions

This study analyzed ZEN’s effects on selected blood coagulation and fibrinolytic parameters. The analysis was based on platelet counts, an indicator of primary hemostasis, as well as an examination of indicators of secondary hemostasis. ZEN’s influence on selected indicators of endothelial cell function (von Willebrand factor, NO levels) and the oxidative status of gilts exposed to ZEN (catalase activity in the blood plasma) were also evaluated. The results indicate that ZEN did not affect the blood coagulation cascade. However, in view of ZEN’s proinflammatory and pro-oxidative effects, the results suggest that this mycotoxin may induce a transient imbalance in the vascular system by influencing vascular endothelial cells. Further research is needed to determine the extent to which exposure to low doses of ZEN affects vascular function, not only at the systemic level, but also in selected tissues and organs where changes in vascular parameters can affect the health and performance of gilts.

## 5. Materials and Methods

This study is a part of project which aimed to assess the effects of feed-borne exposure to ZEN on health status of sexually immature gilts (project No. N N308 628938; Polish National Science Centre). Whereas our previous report described the influence of ZEN on haematological and biochemical indices in blood of the pigs [84], the present study focuses on selected elements of their hemostasis.

### 5.1. Animals and Diet

The study was performed on 18 clinical healthy, dewormed female Polish Large White pigs (19 ± 1.5 kg b.w.) obtained from a commercial fattening farm in Baldy, Poland. The experiment on animals was approved by the Local Ethics Committee affiliated with the National Ethics Committee for Animal Experimentation of the Polish Ministry of Science and Higher Education (Resolution No. 24/2009 issued on 10 July 2009).

Gilts were housed in pens with ad libitum access to water. A feeding program was implemented for 42 days, and porcine diets were formulated in accordance with the nutritional requirements of gilts. Pigs were fed a basal diet containing commercial all-mash feed for piglets with a known composition (Table 4). To eliminate the risk of accidental mycotoxin contamination, feed was tested before and at the end of the feeding experiment for the presence of the following substances: aflatoxin B1, T-2 toxin, ochratoxin A, ZEN, alpha-zearalenol and deoxynivalenol. The above contaminants were identified via common separation techniques with the use of immunoaffinity columns, a high-performance liquid chromatography system (Agilent Technologies, Santa Clara, CA, USA, type 1050 and 1100) with fluorescence and/or ultraviolet detection techniques. None of the tested substances were detected in the analyzed feed.

Animals were selected based on body weight; they were randomly allocated to two treatment groups and exposed to ZEN for 14, 28 and 28 days with a 7-day adaptation period. The control group (control, 9 gilts per pen, 3 gilts for every exposure period) was fed the basal diet with empty gelatin capsules. The experimental group (ZEN, 9 gilts per pen, 3 gilts for every exposure period) was administered the basal diet supplemented with purified ZEN (Sigma Aldrich, Saint Louis, MO, USA) in gelatin capsules that were formulated in accordance with the procedure described by Obremski et al. [93]. The experimental group was administered ZEN at a dose of 5.0 µg·kg^−1^ b.w.·day^−1^. The ZEN dose was selected based on the guidance values for ZEN in complete feeding stuffs for piglets [51]. In both groups, the capsules were administered individually per os, once daily before the morning feeding (one capsule a day per each animal). Weight gains in piglets and feed consumption were monitored once a week to adjust the dose of ZEN. General health status of the gilts was verified via a clinical, biochemical and haematological blood test. Clinical observations were performed twice a day during feeding (at 08:00 and 17:00) to determine the animals’ behavior (activity levels, apathy, interactions within the group), gastrointestinal health (appetite, diarrhea, vomiting, macroscopic analyses of feces consistency) and respiratory health (cough, breathing difficulty, discharge from nostrils and/or conjunctival sac). During the entire experiment, we did not observe any adverse events that would require the isolation and observation of selected animals, treatment or monitoring of vital signs. Clinical signs of hyperestrogenism were not observed.

### 5.2. Tissue Sampling and Preparation for Analyses

Gilts were fasted (12 h) at the end of each exposure period (14, 28 and 42 days). Blood was collected from the anterior vena cava of control (*n* = 3) and treated (*n* = 3) pigs, and individual samples were collected into test tubes treated with ethylenediaminetetraacetic acid (EDTA) or 3.2% sodium citrate. After the collection of blood samples, control (*n* = 3) and treated (*n* = 3) pigs were euthanized at the end of each exposure period according to Resolution No. 24/2009 of the Local Ethics Committee, affiliated with the National Ethics Committee for Animal Experimentation of the Polish Ministry of Science and Higher Education, of 10 July 2009.

### 5.3. Hematological Analyses

The analysis was based on platelet counts as an indicator of primary hemostasis. Platelet counts and mean platelet volume were determined in blood samples collected in test tubes with the addition of K_2_EDTA (2 mL, MEDLAB, Poznan, Poland). The samples were analyzed immediately after collection. The assay was performed in the ADVIA 2021i hematology analyzer (Siemens, Chicago, IL, USA) using laser-based flow cytometry. Blood samples were analyzed individually.

### 5.4. Coagulation Analyses

The blood coagulation system was evaluated based on parameters denoting the activated coagulation cascade pathway. The extrinsic coagulation pathway was analyzed based on prothrombin time and the intrinsic coagulation pathway based on activated partial thromboplastin time. Thrombin time indicates the body’s ability to convert fibrinogen to fibrin, and it is not influenced by the extrinsic or intrinsic prothrombin activation pathway. Fibrinogen participates in the common coagulation pathway, and D-dimers are the smallest products of fibrin degradation and a very sensitive marker of plasminogen activation. Antithrombin activity was determined to evaluate the degree of activation of the anticoagulant system that limits the progression of the coagulant cascade.

Coagulation and fibrinolytic profiles were determined in platelet-depleted plasma samples from 2000× *g* of blood specimens, which were collected into test tubes with 3.2% sodium citrate and centrifuged for 15 min. The following parameters were determined immediately after plasma separation: prothrombin time, activated partial thromboplastin time, thrombin time, fibrinogen concentrations, D-dimer concentrations and antithrombin activity. Coagulation tests were carried out using the Coag-Chrom 3003 device (Bio-Ksel Ltd., Grudziądz, Poland), and the reagents were supplied by the manufacturer. Blood samples were analyzed individually.

### 5.5. Determination of the Von Willebrand Factor Antigen

The von Willebrand factor is released into the blood in a continuous and controlled manner by factors that stimulate the endothelium or platelets. The content of the von Willebrand factor antigen in citrated porcine plasma was determined in an ELISA test. A 96-well plate (Nunc, Roskilde, Denmark) was coated with the anti-von Willebrand factor antibody (DAKO, Glostrup, Denmark) for 12 h and blocked with 1% bovine serum albumin solution (BSA) (Sigma Aldrich, Saint Louis, MO, USA) in phosphate buffer saline (PBS; 137 mM NaCl, 2.7 mM KCl, 8.1 mM Na_2_HPO4, 1.5 mM KH_2_PO4) for 2 h at 37 °C. Samples diluted in 1% solution of BSA in PBS (1:200 *v*/*v*) were incubated for 1 h at 37 °C, and detection was carried out with labeled antibodies conjugated to horseradish peroxidase and diluted in 1% BSA in PBS (DAKO, Glostrup, Denmark). O-phenylenediamine was used as a substrate (Sigma Aldrich, Saint Louis, MO, USA). Samples were incubated with the substrate solution for 30 min at 37 °C. Absorbance was read at λ = 492 nm wavelength using a Tecan Infinite 200 plate reader (Tecan, Grödig, Austria). Pooled normal plasma in sodium citrate was the reference, and the results were expressed as a percentage of the reference standard. Specimens were analyzed in duplicate.

### 5.6. Determination of NO Content

The content of NO in citrate plasma samples was determined via the modified Griess method using a Sigma Aldrich Nitrite/Nitrate Assay Kit (Sigma Aldrich, Saint Louis, MO, USA). The applied method relies on NO’s instability and tendency to undergo oxidation to nitrate and nitrite in tissues. Nitrite (nitrate III) was determined directly, without modification, with the use of the Griess reagent supplied with the kit. To determine the content of NO, which is oxidized to nitrate V, NO was reduced to nitrites using nitrate reductase and the corresponding cofactor (included). Nitrite concentrations were once again determined using the Griess method, and they were read from the calibration curve in the range of 0 to 100 uM. Measurements were performed on 96-well titer plates (Merck, Darmstadt, Germany). Absorbance was measured at a wavelength of λ = 570 nm with the Tecan Infinite 200 plate reader (Tecan, Grödig, Austria). Specimens were analyzed in duplicate.

### 5.7. Determination of Catalase Activity

Catalase activity in plasma samples was measured with the catalase fluorometric detection kit (ENZO Life Sciences, Lörrach, Germany), and the procedure was optimized according to the manufacturer’s recommendations. Plasma samples (diluted 50-fold) or standard curve samples were aliquoted into 96-well black plates (NUNC, Roskilde, Denmark). A standard curve was derived by diluting an appropriate amount of catalase (Sigma Aldrich, Saint Louis, MO, USA) in 1X reaction buffer which was applied to the plate. Then, 50 μL of 40 μM H_2_O_2_ solution (ENZO Life Sciences, Lörrach, Germany) was added to each well, and the plate was incubated for 45 min at room temperature. A further 100 µL of the Reaction Cocktail (ENZO Life Sciences, Lörrach, Germany) was added to each well, and the plate was incubated in the dark for additional 10 min. After incubation, all samples were analyzed in the Tecan Infinite 200 plate reader (Tecan, Grödig, Austria) at an excitation wavelength of 570 nm. Fluorescence was measured at 600 nm. A standard curve was produced by plotting relative fluorescence units against catalase activity (U/mL) in each standard sample used for the measurement. The concentration of unknown samples was determined via interpolation to the standard curve. The standard curve ranged from 0 up to 4 U/mL.

### 5.8. Statistical Analysis

Data from the blood measurements are presented as the median and min–max values (*n* = 3 for each of the two groups of pigs at each exposure period). The significance of differences in the measurements between the experimental groups of pigs were assessed using Kruskal‒Wallis followed by Dunn’s post hoc test (with Bonferroni correction). All statistical calculations were performed using SPSS Statistics 27 (IBM, Armonk, NY, USA), and differences were considered to be significant at *p* < 0.05.

## Figures and Tables

**Table 1:** Hematological measurements of prepubertal female pigs from the untreated group (control) and the group exposed to zearalenone (ZEN) at 5.0 µg·kg b.w.^−1^ × day^−1^ after 14, 28 and 42 days of the experiment.

Measurement	Control			ZEN			Kruskal‒Wallis Test Statistics
	14 d	28 d	42 d	14 d	28 d	42 d	
PLT [10^9^ × L^−1^]	467.0	385.0	463.0	570.0	560.0	421.0	H(5) = 6.802
	(463.0–584.0)	(296.0–416.0)	(325.0–473.0)	(362.0–659.0)	(466.0–581.0)	(370.0–520.0)	*p* = 0.236
MPV [fL]	9.3	9.2	8.7	9.3	8.7	7.9	H(5) = 9.724
	(9.1–11.2)	(7.9–9.3)	(7.7–9.1)	(9.0–10.1)	(7.9–9.0)	(7.5–8.8)	*p* = 0.083

Abbreviations: platelet count (PLT), mean platelet volume (MPV). Values are the median and min–max values (in parentheses) for groups of *n* = 3 each. The significance of differences in the measurements between the experimental groups of pigs were assessed using Kruskal‒Wallis followed by Dunn’s post hoc test (with Bonferroni correction).

**Table 2:** Blood coagulation measurements of prepubertal female pigs from the untreated group (control) and the group exposed to zearalenone (ZEN) at 5.0 µg·kg b.w.^−1^ × day^−1^ after 14, 28, and 42 days of the experiment.

Measurement	Control			ZEN			Kruskal-Wallis Test Statistics
	14 d	28 d	42 d	14 d	28 d	42 d	
PT [s]	15.8	14.7	15.1	16.3	16.3	14.7	H(5) = 6.722
	(15.8–18.8)	(14.5–18.3)	(14.7–16.4)	(15.4–18.4)	(15.9–18.4)	(14.5–15.7)	*p* = 0.242
PT [%]	84.0	90.0	88.0	82.0	82.0	90.0	H(5) = 5.833
	(71.0–84.0)	(72.0–91.0)	(81.0–90.0)	(72.0–86.0)	(72.0–83.0)	(84.0–91.0)	*p* = 0.323
APTT [s]	36.1	36.1	35.5	35.6	38.2	34.7	H(5) = 2.074
	(31.0–38.2)	(31.7–38.7)	(35.3–42.0)	(28.0–45.6)	(35.4–43.2)	(34.5–35.8)	*p* = 0.839
FBG [g × L^−1^]	2.7	3.4	3.0	3.8	4.6	3.0	H(5) = 3.573
	(2.3–3.3)	(2.8–3.6)	(2.9–3.1)	(2.2–5.8)	(2.9–4.8)	(2.9–3.1)	*p* = 0.612
TT [s]	29.9	26.7	29.2	25.8	25.0	27.3	H(5) = 10.103
	(29.7–36.1)	(26.1–32.3)	(22.4–31.7)	(22.1–26.1)	(23.1–25.6)	(27.3–28.2)	*p* = 0.072
AT III [%]	81.0	99.0	97.0	95.0	92.0	98.0	H(5) = 1.834
	(78.0–135.0)	(92.0–107.0)	(96.0–104.0)	(79.0–105.0)	(91.0–101.0)	(96.0–100.0)	*p* = 0.872
D-D [µg × L^−1^]	453.0	399.0	349.0	398.0	489.0	352.0	H(5) = 3.015
	(289.0–453.0)	(294.0–404.0)	(324.0–358.0)	(255.0–602.0)	(344.0–567.0)	(275.0–355.0)	*p* = 0.698

Abbreviations: prothrombin time (PT), activated partial thromboplastin time (APTT), fibrinogen (FBG), thrombin time (TT), antithrombin III (AT III), d-dimers (D-D). Values are the median, and min–max values (in parentheses) for groups of *n* = 3 each. The significance of differences in the measurements between the experimental groups of pigs were assessed using Kruskal-Wallis followed by Dunn’s post hoc test (with Bonferroni correction).

**Table 3:** Concentration of the von Willebrand factor (vWf), nitric oxide (NO) levels and catalase activity (CAT) in the plasma of prepubertal female pigs from the untreated group (control) and the group exposed to zearalenone (ZEN) at 5.0 µg × kg b.w.^−1^·day^−1^ after 14, 28 and 42 days of the experiment.

Measurement	Control			ZEN			Kruskal-Wallis Test Statistics
	14 d	28 d	42 d	14 d	28 d	42 d	
vWf [%]	92.6 ^ab^	117.4 ^ab^	98.3 ^ab^	127.1 ^a^	119.3 ^ab^	84.6 ^b^	H(5) = 11.386
	(91.7–136.6)	(116.4–119.9)	(95.7–99.5)	(126.0–131.4)	(114.9–131.3)	(78.8–87.0)	*p* = 0.044
NO [µmol × L^−1^]	190.3	172.2	143.3	187.0	130.2	146.6	H(5) = 13.164
	(179.3–194.5)	(171.6–196.8)	(116.6–158.7)	(177.8–188.7)	(116.3–154.6)	(119.3–149.5)	*p* = 0.022
CAT [U × mL^−1^]	14.6 ^c^	30.6 ^ab^	32.2 ^a^	18.9 ^abc^	18.0 ^abc^	21.5 ^abc^	H(5) = 13.959
	(9.7–15.9)	(30.4–35.3)	(22.8–34.4)	(18.0–20.6)	(17.5–23.6)	(18.7–21.8)	*p* = 0.016

Values are the median and min–max values (in parentheses) for groups of *n* = 3 each. Different letters denote significant differences in the measurements between the experimental groups of pigs, which were assessed using Kruskal‒Wallis followed by Dunn’s post hoc test (with Bonferroni correction).

**Table 4:** Feed parameters per 1 kg of the product.

Parameter	Unit	Content
Metabolizable energy	MJ × kg^−1^	13.15
Net energy	kcal × kg^−1^	2260
Total protein	%	16.5
Crude fat	%	2.3
Crude fiber	max %	3.4
Lysine	%	1.27
Methionine	%	0.35
Threonine	%	0.72
Tryptophan	%	0.22
Calcium	%	0.73
Phosphorus	%	0.54
Sodium	%	0.19
Calcium	min %	0.73
Total phosphorus	min %	0.54
Vitamin A	IU	15,000
Vitamin D_3_	IU	2000
Vitamin E	mg∙kg^−1^	100
Phytase	present	+
Enzymatic preparation	present	+
Flavoring	present	+
Acidifiers (preservatives)	present	+

## Data Availability

Not applicable.

## Abbreviations

BSA: bovine serum albumin, eNOS: endothelial nitric oxide synthase, ELISA: enzyme-linked immunosorbent assay, ERs: estrogen receptors, EDTA: ethylenediaminetetraacetic acid, K2EDTA: ethylenediaminetetraacetic acid dipotassium salt, IL: interleukin, LOAEL: lowest observable adverse effect level, NADPH: nicotinamide adenine dinucleotide phosphate, NO: nitric oxide, NOS: nitric oxide synthase, NOAEL: no observable adverse effect level, PBS: phosphate buffer saline, ROS: reactive oxygen species, vWf: von Willebrand factor: vWf, ZEN: zearalenone.
